# Pertussis Prevention: Reasons for Resurgence, and Differences in the Current Acellular Pertussis Vaccines

**DOI:** 10.3389/fimmu.2019.01344

**Published:** 2019-07-03

**Authors:** Susanna Esposito, Paola Stefanelli, Norman K. Fry, Giorgio Fedele, Qiushui He, Pauline Paterson, Tina Tan, Markus Knuf, Carlos Rodrigo, Catherine Weil Olivier, Katie L. Flanagan, Ivan Hung, Iria Lutsar, Kathryn Edwards, Miguel O'Ryan, Nicola Principi, World Association of Infectious Diseases and Immunological Disorders (WAidid) and the Vaccine Study Group of the European Society of Clinical Microbiology and Infectious Diseases (EVASG)

**Affiliations:** ^1^Department of Surgical and Biomedical Sciences, Paediatric Clinic, Università degli Studi di Perugia, Perugia, Italy; ^2^Department of Infectious Diseases, Istituto Superiore di Sanità, Rome, Italy; ^3^Immunisation and Countermeasures Division, Public Health England–National Infection Service, London, United Kingdom; ^4^Institute of Biomedicine, University of Turku, Turku, Finland; ^5^Department of Medical Microbiology, Capital Medical University, Beijing, China; ^6^Department of Infectious Disease Epidemiology, The Vaccine Confidence Project TM, London School of Hygiene & Tropical Medicine, London, United Kingdom; ^7^Division of Pediatric Infectious Diseases, Department of Pediatrics, Northwestern University Feinberg School of Medicine, Ann & Robert H. Lurie Children's Hospital of Chicago, Chicago, IL, United States; ^8^Children's Hospital, Helios HSk, Wiesbaden, Germany; ^9^Department of Pediatrics, University Medicine, Mainz, Germany; ^10^Department of Pediatrics, Vall d'Hebron University Hospital, Barcelona, Spain; ^11^School of Medicine-Germans Trias i Pujol University Hospita, Universidad Autónoma de Barcelona, Barcelona, Spain; ^12^Retired, Neuilly-sur-Seine, France; ^13^School of Medicine, College of Health and Medicine, University of Tasmania, Hobart, TAS, Australia; ^14^School of Health and Biomedical Science, RMIT University, Melbourne, VIC, Australia; ^15^Department of Immunology and Pathology, Monash University, Melbourne, VIC, Australia; ^16^Department of Medicine, LKS Faculty of Medicine, The University of Hong Kong, Hong Kong, China; ^17^Department of Microbiology, Institute of Biomedicine and Translational Medicine, University of Tartu, Tartu, Estonia; ^18^Division of Pediatric Infectious Diseases, Department of Pediatrics, Vanderbilt University School of Medicine, Nashville, TN, United States; ^19^Microbiology and Mycology Program, Faculty of Medicine, Institute of Immunology and Immunotherapy, University of Chile, Santiago, Chile; ^20^Retired, Milan, Italy

**Keywords:** acellular pertussis vaccine, *Bordetella pertussis*, pertussis, whole-cell pertussis vaccine, pertussis prevention

## Abstract

Pertussis is an acute respiratory disease caused by *Bordetella pertussis*. Due to its frequency and severity, prevention of pertussis has been considered an important public health issue for many years. The development of the whole-cell pertussis vaccine (wPV) and its introduction into the pediatric immunization schedule was associated with a marked reduction in pertussis cases in the vaccinated cohort. However, due to the frequency of local and systemic adverse events after immunization with wPV, work on a less reactive vaccine was undertaken based on isolated *B. pertussis* components that induced protective immune responses with fewer local and systemic reactions. These component vaccines were termed acellular vaccines and contained one or more pertussis antigens, including pertussis toxin (PT), filamentous haemagglutinin (FHA), pertactin (PRN), and fimbrial proteins 2 (FIM2) and 3 (FIM3). Preparations containing up to five components were developed, and several efficacy trials clearly demonstrated that the aPVs were able to confer comparable short-term protection than the most effective wPVs with fewer local and systemic reactions. There has been a resurgence of pertussis observed in recent years. This paper reports the results of a Consensus Conference organized by the World Association for Infectious Disease and Immunological Disorders (WAidid) on June 22, 2018, in Perugia, Italy, with the goal of evaluating the most important reasons for the pertussis resurgence and the role of different aPVs in this resurgence.

## Introduction

Pertussis is an acute respiratory disease caused by *Bordetella pertussis*, a Gram-negative bacterial pathogen (1). Before the availability of vaccines, pertussis was a frequent cause of morbidity and mortality, particularly in infants and young children. The introduction of pertussis-containing vaccines into the immunization schedule of infants and children has reduced pertussis incidence, although sporadic outbreaks remain relatively common. In a recent publication (2), it was projected that in 2014, 24. One million cases of pertussis occurred around the world in children aged <5 years with ~160,000 deaths and many hospitalization admissions, some to pediatric intensive care units. Due to its frequency and severity, pertussis prevention has been considered an important public health issue for many years. The development of the whole-cell pertussis vaccine (wPV) and its introduction into the pediatric immunization schedule was associated with a marked reduction in pertussis cases in the vaccinated cohort (3). With widespread use of wPV, reporting of pertussis declined significantly worldwide, and both hospitalization rates and deaths due to pertussis were greatly reduced. However, due to the frequency of local and systemic adverse events after immunization with wPV, many parents were refusing vaccination for their children and lawsuits against the vaccine manufacturers were forcing many of them to stop producing the vaccine (4–6). Currently, wPV is no longer being used in most developed countries, but remains in use in most low and middle income countries (LMIC) (4, 7–9).

**Figure 1:**
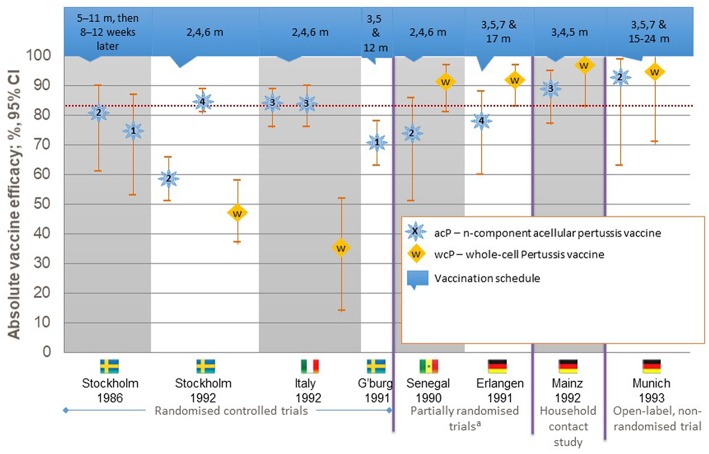
Efficacy of pertussis vaccines against World Health Organization-defined typical pertussis.

Work on a less reactive vaccine was undertaken based on the isolation of *B. pertussis* components that induced protective immune responses with fewer local and systemic reactions. These component vaccines were termed acellular vaccines and were composed of one or more pertussis antigens, including pertussis toxin (PT), filamentous haemagglutinin (FHA), pertactin (PRN), and fimbrial proteins 2 (FIM2) and 3 (FIM3). Preparations containing up to five components were developed, and several efficacy trials clearly demonstrated that the aPVs were able to confer comparable short-term protection to the most effective wPVs with fewer local and systemic reactions (10–15) (Figure 1). With this enhanced safety profile and despite being more expensive than wPVs, aPVs were included in the pediatric immunization schedules of many countries, particularly in the industrialized world (16, 17). After over a decade of use, a rise in pertussis incidence was demonstrated in several industrialized countries, including Australia and the United States (18–22). Some of this apparent increase may also be due to improved diagnostic methods, such as the use of molecular techniques to diagnose pertussis, but most experts think that it is also a result of more rapid waning of immunity after immunization with the acellular vaccines. Data from the United States document that 4,000 cases were reported annually in the 1980s, but increases to 25,827, 25,616, 27,500, and 48,277 cases were reported in 2004, 2005, 2010, and 2012, respectively (19–21). In 2016, 17,972 cases were reported, with an incidence rate of 70.9 per 100,000 in children <6 months, 31.9 per 100 000 in 6 to 11 month-olds, and 13.7, 14.8, and 16.3 for those aged 1–6, 7–10, and 11–19 years, respectively (23). However, the under-reporting of mild cases would be lower these rates.

Trends that are not substantially different have been registered in other areas. In Europe, 48,446 pertussis cases were reported to the European Surveillance System by 30 EU/EEA countries in 2016. This number was slightly higher than that reported in 2012 (42,572), the year of peak pertussis incidence in Europe. As in the USA, rates were higher among children <1 year. In this younger group, the notification rate was 73.6 cases per 100,000 population, a value significantly higher than that reported in 2014 (51.6 per 100,000 population). Moreover, children between 10 and 14 years of age were reported to have the second highest incidence rates of pertussis, ~30 cases per 100,000 population (24).

The reasons for pertussis resurgence have been investigated, and several possibilities have been considered. This paper reports the results of a Consensus Conference organized by the World Association for Infectious Disease and Immunological Disorders (WAidid) on June 22, 2018, in Perugia, Italy, with the goal of evaluating the most important reasons for the pertussis resurgence and the role of different aPVs in this resurgence.

## Laboratory Methods for Pertussis Diagnosis

**Figure 2:**
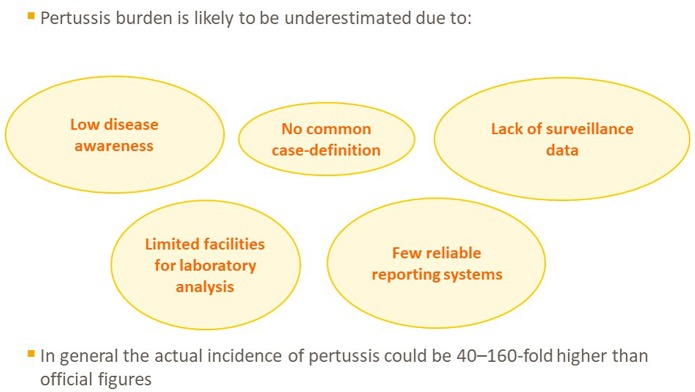
Reasons for underestimation of pertussis incidence and mortality.

Several reports indicate that resurgence of pertussis might be due, at least in part, to an artifact, resulting from an incomplete identification of pertussis cases in the past (Figure 2). The sole use of cultures to confirm *B. pertussis* infection has contributed to this phenomenon. Although the culture method has 100% specificity, its sensitivity is very low (25). Moreover, nasopharyngeal samples for culture have to be collected within the first 15 days of the disease, when symptoms are frequently non-specific, and such a diagnosis is rarely suspected. Furthermore, isolation can become difficult if the patient has recently been treated with antibiotics that are active against *B. pertussis* (26). In addition, pertussis cases occurring outside the first year of age (including in adulthood) frequently present as only prolonged cough and are undiagnosed, resulting in an underestimation of the true disease incidence (25, 26).

In recent years, more attention has been given to the epidemiology of pertussis disease in all age groups. New criteria for the clinical definition of pertussis according to age have been suggested (27). The enhanced identification of pertussis cases using molecular methods, particularly in adolescents and adults, has increased the total number of reported cases significantly (28). The molecular techniques, particularly real-time-polymerase chain reaction (RT-PCR), are widely available in clinical practice and can be completed in a few hours, allowing rapid diagnosis of pertussis. Moreover, since the techniques do not require viable bacteria, they are more sensitive (70–99% vs. 12–60%). However, similar to culture, they are maximally sensitive during the first 2–3 weeks of disease (29). Finally, to confirm *B. pertussis* infection beyond this period in previously undiagnosed patients, serologic methods detecting anti-pertussis toxin IgG in serum and saliva have been developed and validated (30).

The availability of reliable tests for pertussis diagnosis to physicians was rapidly associated with a relevant increase in their use and in the number of pertussis cases being reported. In Australia, the use of both both PCR and serology in patients with pertussis-like symptoms increased from 0.5% in the period from April 2000 to March 2004 to 1.7% in the period between March 2010 and March 2011. At the same time in Australia, the proportion of pertussis notifications with a PCR-confirmed diagnosis increased from 16.3% in the period from April 2000 to March 2004 to 65.3% in the period between April 2010 and March 2011 (31). In Canada, the availability of a more sensitive PCR assay was associated with a concomitant 6-fold increase in specimen submissions and a 5-fold increase in pertussis incidence (32).

Assessing the impact of the enhanced diagnostic methods to the resurgence of disease is difficult, given the periodicity of naturally occurring pertussis, the different control measures and reporting standards for different countries, and the different vaccines and schedules recommended in each country. However, a study carried out by a working group of the WHO seems to indicate that although improved diagnosis may play a role, a real increase in pertussis incidence has occurred in recent years (33). The group prepared a questionnaire to obtain information on pertussis incidence, vaccination coverage and schedules, surveillance methods, case definitions, and the type of vaccine used. The questionnaire was widely distributed to countries thought to have achieved long-standing high vaccine coverage and effective disease control. Resurgence was defined as evidence of a burden of disease higher than that expected when compared to previous reporting cycles in the same setting. A total of 19 countries participated. Although the increased pertussis incidence could be ascribed to cyclic patterns amplified by detection bias in most of these countries, in five of them (Australia, Chile, Portugal, USA, and UK), the presence of a true resurgence of pertussis in recent years was clearly demonstrated. Only one country using a wPV, Chile, reported a resurgence. However, this resurgence was mainly ascribed to decreased of vaccine coverage, variable coverage within the various districts, changes in the surveillance practices, and problems with the specificity of diagnostic tests. The WHO group concluded that pertussis resurgence was not observed in any country using wPVs, suggesting that a link between aPV use and increased pertussis reporting existed (33).

## Reasons for Pertussis Resurgence After Acellular Pertussis Vaccine Introduction

### Waning of Immunity

As is the case with immunization, natural *B. pertussis* infection does not assure permanent protection against pertussis. Several studies have documented that a second episode of pertussis can occur some years after the first one. Current estimates of the duration of protection due to natural infection range from 7 to 10 years (34, 35) to 20 years (36), but there is evidence that it can be as short as 3.5 years (37). Differences may be due to methods used to evaluate protection and pertussis epidemiology in different countries. However, duration of protection due to natural *B. pertussis* infection can vary from subject to subject as evidenced by Wearing and Rohani (38) who, exploring the inter-epidemic period and fade-out-frequency with a mathematical model, concluded that more than 10% of individuals lose protection within 10 years from infection whereas others are protected for more than 30 years.

A similar or only slightly reduced duration of protection has been calculated after immunization with wPV. Lambert (39) studied a pertussis outbreak that occurred in 1962 in Kent County, Michigan, USA. He found that the incidence of pertussis in vaccinated persons was directly related to the interval since the last immunization with wPV. Among the 210 vaccinated individuals with pertussis, attack rates were 21, 47, and 65% in groups of people who had received the vaccine within the last 4, 4 to 7 years earlier and 8–11 years earlier, respectively. Jenkinson (40) conducted a 10 year study of pertussis in a discrete general practice community in the UK and reported that protection due to immunization with wPV was still effective in 85% of children 4 years after immunization, but was reduced to 50% in the following 3 years.

In contrast, the duration of immunity after immunization with aPVs appears to be shorter, independent of the schedule used, the numbers, and concentrations of antigens included in each vaccine and the methods used to prepare the vaccines. Reports also suggested that pertussis occurred significantly earlier in subjects fully vaccinated with aPV than in those given wPV (41–43). Clark et al. (41) in 2012 reported that children who were fully immunized during infancy with an aPV had pertussis more often in the first years of school, while those given a wPV were at higher risk later, mainly during adolescence. Similar differences were demonstrated by Vickers et al. (43), who found that children who had received an aPV during infancy were already at risk of pertussis within the first 4 years of life, whereas those vaccinated with wPV did not contract pertussis until 5–9 years. Finally, Klein et al. (44) demonstrated that most of the pertussis cases diagnosed in adolescents during an outbreak were seen in individuals fully immunized during infancy with an aPV, rather than in those who had received wPV. Individuals receiving only aPV had five times higher odds of pertussis disease than those receiving wPV (OR 5.63, 95% CI 2.55–12.46). When aPV effectiveness (VE) was measured over time, its effectiveness was lower than that expected after wPV or natural infection (44). Early waning of immunity was reported regardless of the schedule used for immunization (44).

The addition of booster doses of aPV to prolong protection was also assessed. Although there was transient protection afforded by additional booster doses, the protection waned rapidly. A meta-analysis of 11 studies (45) that measured long-term immunity to pertussis after three or five doses of diphtheria-tetanus-aP (DTaP), according to the schedules used in many European countries and in the USA, respectively, did not reveal a significant difference between the annual odds of pertussis for the three or five dose regimens. It was calculated that for every additional year after the last dose of DTaP, the risk of pertussis increased 1.33 times (95% CI 1.23–1.43), leading to the conclusion that 8.5 years after the last aPV dose, only 10% of children were still protected against disease. Similar results were reported in another meta-analysis including studies of aPVs administered according to the USA schedule (46). VE was compared after the childhood series (five doses) and after an adolescent booster dose (sixth dose). Relative VE was defined as VE in the population given prior doses of an aPV and absolute VE was defined as VE in an aPV-naïve population. Absolute VE after the childhood series was 91% (95% CI 87–95%) but declined annually by 9.6% (46). Initial relative VE after adolescent boosting was 70% (95% CI: 54 to 86%) and declined by 45.3% annually. The absolute VE of the full six-dose aPV series was estimated to be 85% (95% CI: 84–86%) in the first year after series completion. However, it declined by 11.7% (95% CI: 11.1 to 12.3%) per year, and at 18 years of age, protection was limited to 28.2% of immunized patients (95% CI: 27 to 29%) (46).

Wang et al. (47) studied 279 children aged 5 to 15 years who presented to primary care with a persistent cough of 2 to 8 weeks duration. Evidence of recent *B. pertussis* infection based on a high oral fluid anti-pertussis toxin IgG titer was demonstrated in 215 children who had been fully vaccinated. Risk was higher in those who had been immunized ≥7 years earlier, but in 12% of these cases, chronic cough was demonstrated in patients given an aPV <7 years before. Further evidence of waning immunity after recent aPV immunization was reported by Principi et al. (48) who documented *B. pertussis* infection in 18.7% (95% CI 11.5–28.0) of children and adolescents with chronic cough who had been immunized with an aPV a few years previously (<2 years in some cases).

### Immune Responses to Pertussis Vaccines and Natural Infection

Studies that have compared immune responses after natural *B. pertussis* infection and the administration of both wPVs and aPVs have clearly shown that the immune stimulation evoked by aPVs is different from that due to natural infection and wPVs (49–51). Natural infection evokes both mucosal and systemic immune responses, while aPVs induce only a systemic immune response. As *B*. pertussis is a mucosal pathogen and only exceptionally causes infection outside the respiratory tract, this difference is of particular importance in pertussis control. Mucosal immunity is essential to prevent colonization and transmission of *B. pertussis* organisms. Consequently, preventive measures such as aPVs that do not induce a valid mucosal response can prevent disease but cannot avoid infection and transmission. Animal studies have shown that natural infection is associated with a strong secretory IgA response in both the upper and lower airways and induction of resident memory T cells (TRM) (52, 53). Moreover, it has been recently reported (54) that IL-17 and IFN-γ-secreting CD69+CD4+ TRM cells were expanded in the respiratory tract after *B. pertussis* challenge of mice immunized with wP, but not aP vaccines. However, natural infection was associated with the most persistent protection against nasal colonization and this correlated with potent induction of nasal tissue TRM cells. These animal data suggest that the lack of mucosal immune response after aPV administration might explain its lower efficacy when compared to wPVs and the shorter duration of protection compared to both wPV vaccination and natural infection.

Clear differences between systemic immune response after natural infection and aP and wP vaccines. Natural infection and wPvs induce antibodies of the IgG1, IgG2, and IgG3 subclasses, with marginal production of IgG4 (55), suggesting a strong Th1 response. In contrast, the immune response after aPVs evoke a mixed Th2 and Th17 response (56). APVs evoke the production of IgG1 and IgG4 antibodies, which is consistent with a Th2 response. Furthermore, aPVs evoke CD4+ T-cells that produce high concentrations of IL-4 and IL-5 and low amounts of IFNγ, again consistent with a Th2 response (57).

Since Th1 cytokines play an important role in protection against pertussis (58, 59), this finding can further explain the better protection offered by wPVs and natural infection. Studies carried out in children who have received infant series of either wPV or aPVs have shown children given aPVs exhibited higher pertussis-specific antibody levels and higher memory B- and T-cell responses (5, 60–63). Although no correlates of antibody protection for pertussis have been established (64), the higher IgG levels in aPV-immunized children could lead to the conclusion that better humoral protection was afforded by the aP rather than wP vaccines. However, the antigens measured were only those included in the aPVs and not the additional antigens included in the wPs.

These differences in immune responses persist over time, even after booster aPVs (65, 66). The administration of aPV booster doses at 4 and 9 years of age was associated with an increase in the production of IgG4, regardless of the type of vaccine used for priming, but was significantly higher in aPV-primed children (66). IgG4 antibodies are unable to activate the complement system and lead to a suboptimal inflammatory response with impaired phagocytosis and antimicrobial defense, another potential mechanism for the lower efficacy of aPVs compared to wPVs (67). Moreover, the evidence that production of IgG4 after immunization with aPV increases with each dose seems to indicate that the protection offered by aPVs tends to be as shorter with each subsequent boosters (68, 69). Preadolescent booster vaccination with an aPV was found to induce lower B-cell and Th1 cell responses in aPV-primed compared with wPV-primed children, resulting in significantly lower Th1/Th2 ratios. Confirming this, it has been shown that wPv or aPV primary immunizations in infancy determines adolescent cellular immune profiles, showing a beneficial Th1-dominated response after wP-priming (69). These findings of a preferential Th1 response were also shown in the baboon model, with aPV vaccines preventing disease after natural pertussis challenge, but not preventing transmission of pertussis organisms (70). All these findings indicate that although aPVs are as individually protective as wPVs in the first years after priming, they induce shorter long-term protection than wPVs and a different profile of pertussis-specific immunity.

Finally, aPV pertussis vaccines do not prevent colonization. Consequently, they do not reduce the circulation of *B. pertussis* and do not exert any herd immunity effect. These findings at least partly explain the resurgence of pertussis.

### Genetic Modifications of *Bordetella pertussis*

Circulation of *B. pertussis* strains with modified or absent antigens included in the aPV have been reported in both the pre-vaccine era and the aPV era (71–73). Moreover, strains with polymorphisms of the PT gene resulting in the production of greater amounts of this protein have been detected (74–79). Although it cannot be excluded that this phenomenon might simply be derived from the natural evolutionary course of *B. pertussis*, it has been proposed that it might be a consequence of *B. pertussis* adaptation to aPV use (80).

Genes encoding antigens included in the aPV vaccines have evolved at higher rates than other non-vaccine surface protein-encoding genes soon after the introduction of aPVs into the pediatric immunization schedule (81). The most compelling data have been the evolution of PRN-negative *B. pertussis* strains according to the use of vaccines PRN-containing vaccines. With some exceptions (82–84), studies have demonstrated that the emergence of PRN-deficient strains has resulted as a consequence of aPV-induced selection pressure. The rate of PRN-negative isolates is significantly correlated with aPV use in the USA (85). In Denmark, where an aPV without PRN is used, no PRN-deficient isolates have been detected (86). In Japan where aPVs with PRN were administered for many years (87), consistent rates of PRN-negative strains have been demonstrated over time (2005–2007, 41%; 2008–2010, 35%; and 2011–2013, 25%) (88, 89). However, when these aPV vaccines were replaced with a preparation without PRN in November 2012, a marked reduction of PRN-deleted strains was observed (2014–2016, 8%) (90). The clinical relevance of PRN-deleted strains has not been precisely defined (80), but children infected with these strains do not have more severe pertussis (91, 92), In contrast, *B. pertussis* strains with the enhanced PT promoter allele PTP3, instead of the common PTP2 allele, were found to produce greater amounts of PT (74) and cause more severe disease in younger infants (92).

Interesting, *B. pertussis* strains lacking the *PRN* gene show increased fitness and/or prolonged infection times in animals immunized with ACVs (74, 93, 94). This finding suggests that loss of PRN could lead to a reduced immune response to aPVs and favor pertussis resurgence. However, clinical studies that have evaluated the effectiveness of aPVs containing PRN in the setting of PRN-deficient pertussis have produced conflicting results. One study in the US (80) assessed the VEs of a five-dose DTaP series among 4–10 year-olds and a Tdap booster among 11–19 year-olds in an area where >90% of *B. pertussis* strains were PRN deficient. It was found that overall DTaP VE was 84% (95% CI 58–94%) while that of TdaP was 70% (95% CI 54–81%), which do not substantially differ than rates reported during the circulation of PRN-positive strains. In contrast, a second US study revealed that in vaccinated persons, the likelihood of suffering from pertussis disease was greater if the infecting strain was PRN-negative than if it is PRN-positive (85).

In conclusion, aPV use seems to favor adaptation of *B. pertussis* strains with emergence of mutated strains. However, the role of genetic modification in reducing aPV protection remains unclear with future studies needed.

## Role of Antigens Included in Presently Available Vaccines in Conditioning Protection

Although pertussis resurgence has been demonstrated to be independent of the type of aPV used, it is theoretically possible that the composition of vaccines and the immunization strategies may have played a role in modifying the pertussis incidence. However, estimates of aPV efficacy and comparisons between different aPVs are very problematic for several reasons. First, the criteria for the diagnosis of pertussis used in the various aPV effectiveness trials have not been uniform. In some cases, significant underestimations of the real pertussis incidence may have limited the reliability of final results. When the WHO's clinical case definition of pertussis as prolonged paroxysmal cough is used, it is highly likely that most of the mild cases are not included. Second, study designs, administration schedules, and duration of follow-up have not been consistent in the effectiveness trials. In many European countries, the primary series includes only two doses of an aPV with a booster dose at ~1 year. In contrast, in other countries, including the US, the primary series is based on three doses within the first 6 months of life, with a booster dose given after the first birthday. Third, most, but not all, national immunization schedules include a booster before entering school and during adolescence. Fourth, the composition of the administered aPV can vary. Most of these studies have been carried out with vaccines containing three or five antigens, but in earlier studies vaccines with only PT have been included. In addition, the quantity of antigen can differ among the preparations. For example, GSK DTaP vaccines contain 25 μg PT, 25 μg FHA, and 8 μg PRN, while the Sanofi preparation also includes FIM2 and three different amounts of PT, FHA, and PRN for the primary and booster doses. Tdap contains 10 μg PT, 5 μg FHA, and 3 μg PRN when administered alone, but when Tdap is combined with polio, hepatitis B, and *Haemophilus influenzae* type b, the PT and FHA content is increased to 20 μg. In addition, the type of aluminum salt used as an adjuvant and its content vary slightly among between vaccines. Finally, there has been no single study that directly compares all aPV vaccines with different numbers and quantities of included antigens.

### Role of the Number of Pertussis Antigens

In those studies that directly compared vaccines using similar vaccine schedules, similar definitions of pertussis disease, and comparable durations of follow-up, it can be concluded that the 3-component aPV (3aPV) and the 5-component aPV (5aPV) have comparable efficacy. Greco et al. evaluated two 3aPVs produced by different pharmaceutical companies (12), and Gustafsson et al. (13) studied a 5aPV, with both studies being conducted in children that had received three doses at ~2, 4, and 6 months of age and were followed for 2 years. For both 3aPVs, the overall efficacy was 84% (95% CI 75.8–89.4 for the first and 76.2–89.7 for the second 3aPV), while that of the 5aPV was 85.2% (95% CI 80.6–88.8) 1 year after first dose (13).

All these vaccines contain PT, which might explain these relatively similar results. PT seems to be essential for conferring protection (95, 96). In Sweden (10) and Denmark (97) a vaccine containing only PT demonstrated effectiveness that did not differ from that of multivalent aPVs concomitantly used in other countries. However, lower levels of anti-PT antibodies have been associated with increased susceptibility to *B. pertussis* infection (95, 96). It has not been determined which type and quantity of PT is able to confer the greatest short- and long-term protection. Because of its various noxious effects, PT must be detoxified before inclusion in aPVs. Detoxification can be achieved genetically or through chemical treatment. The results of these methods are quite different, as genetic detoxification does not modify the antigenic characteristics of PT and leads to a protein with superior immunogenicity to chemically detoxified PT, as clearly demonstrated by an efficacy trial showing that the PT-9K/129G-based vaccine induces earlier and longer-lasting protection (98). Additionally, lower doses of genetically detoxified PT seem to stimulate comparable protection as higher doses of the chemically detoxified product. In the study by Greco et al. (12) in which two 3aPVs were evaluated, the protection offered by the two vaccines was comparable but was achieved with 5 μg of genetically detoxified PT and with 25 μg of chemically detoxified PT.

Finally, it cannot be forgotten that not all chemical treatments have the same impact on PT. Detoxification with formaldehyde, glutaraldehyde, or with combined procedures are more destructive of epitopes than detoxification with hydrogen peroxide, evoking a lower immune response (50). Confirming this notion, important differences have been evidenced when comparing two aPVs detoxified by different chemical procedures in terms of long-term B and T-cell immune responses (99, 100).

The relevance of FHA for inducing protection is less clear. Several studies in mice suggest that pre-existing serum anti-FHA antibodies do not protect against pertussis in mice (101). FHA antibody induced by passive or active immunization did not protect mice against intracerebral or pulmonary challenge with *B. pertussis* (102). Finally, human studies conducted in Japan suggested that comparable protection was seen after either an aPV containing PT and FHA or only an aPV including only PT, although the results of this study remain debatable, since the PT and FHA combination vaccine contained only approximately 50% of the PT in PT only product. In contrast, another study in humans found that the relative efficacy of a monovalent PT vaccine was significantly less than that seen after combined PT-FHA vaccine during 3 years of passive surveillance (103). However, as FHA is an adhesin that is essential for the adherence of *B. pertussis* to the respiratory epithelium, it has been suggested that mucosal antibodies against this antigen could block adherence, thereby protecting against colonization, and, consequently, disease (104). This hypothesis is supported by the evidence that FHA delivered to experimental animals intranasally can provide protection against aerosol challenge with *B. pertussis* (105, 106).

The importance of PRN has already been discussed, and whether the lack of PRN in the infecting strain can reduce aPV efficacy remains controversial. Although protection against pertussis after aPV administration has been correlated with high serum anti-PRN antibody concentrations, pre-existing PRN antibodies do not protect against *B. pertussis* infection (97). Further studies are in progress to evaluate the impact of circulating *B. pertussis* strains lacking pertactin in a systematic project among EU countries.

Fimbriae are adhesins, and the hypothesis of a potential role reported previously for FHA can also be suggested for Fim2 and Fim3. However, data on the potential ability of these antigens to induce protective mucosal immunity are lacking. Serum levels of antibodies against Fim2 and Fim3 have been correlated with protection after household exposure to *B. pertussis* (107). Moreover, in a clinical trial, when mild cases of pertussis were included in the case definition, the efficacy of a 5aPV was found to be significantly higher than that of a two or three component aPV. Comparing the relative risk of 3aPV recipients acquiring pertussis to those receiving 5aPV was 1.82 (95% CI 1.14–2.90), suggesting a protective role of fimbriae (10, 107). In the presently available vaccines, FIM2 seems to be more important than FIM 3, as demonstrated by data collected during long-term evaluation of children included in a study by Olin et al. (108). A slight but significant reduction in 5aPV efficacy was seen over time when compared to 3aPV. During that time period, the expression of fimbriae in circulating *B. pertussis* changed from predominantly Fim2 to Fim3; suggesting that the stronger immune responses to the Fim2 antigen than the Fim3 antigen in the vaccinated subjects was the cause of the reduced long-term efficacy of the 5aPV, suggesting that FIM 2 evokes more relevant protection after 5aPV administration (109).

## Vaccination Coverage and Vaccine Hesitancy

Although coverage with one dose of diphtheria- tetanus-pertussis vaccine (DTP1) is extremely high globally with overall levels at 90% in 2017, the coverage varies between widely 49 and 99%, depending on the country (110, 111). In 2017, the greatest number of cases of pertussis were reported in in India (23,766), Germany (16,183), Australia (12,114), and China (10,390), countries with national vaccination coverage rates of DTP1 all above 90% and coverage rates for three doses of DTP (DTP3) above 88% (112). However, coverage may be high nationally, there can be regional variation leading to sporadic pertussis cases and disease outbreaks (111).

Pertussis is an important cause of childhood morbidity and mortality, especially in infants under 6 months of age. Maternal pertussis immunization is an effective strategy to protect infants during this vulnerable period, prior to them being protected during their childhood vaccination. Maternal pertussis immunization has been included in national recommendations across several high-income countries, including the United States in 2011, the United Kingdom in 2012, and in Australia in 2015 (113, 114). Reasons for non-vaccination in pregnant women are context specific, and include a lack of awareness, a lack of access, a lack of perceived need for the vaccine, and concerns about its safety and effectiveness (115).

## Conclusions

The resurgence of pertussis observed in recent years seems to be a complex but real phenomenon resulting from a number of cases, including the use of aPV in many locales. Lack of mucosal immune responses after aPV administration favor infection, persistent colonization, and transmission of the pathogen. Moreover, earlier waning of protective immunity and the circulation of *B. pertussis* variants depleted of vaccine-included antigens further favor the increase in pertussis disease. Several different aPVs are available, but it has yet to be determined which of them confers the highest and the most-prolonged protection. Further studies are needed to evaluate the importance of individual antigens included in aPVs in conferring protection against disease, colonization, and transmission. However, present knowledge seems to indicate that PT, particularly if genetically detoxified, represents the main antigen that ensures protection from disease even if not from infection. The contribution of FHA, PRN, and FIM2 and FIM3 in vaccine efficacy and long-lasting protection is still under discussion and needs further study.

The optimal pertussis vaccine would be one that induced both a mucosal and systemic responses similar to those occurring under natural infection, leading to a long-term protection against both disease and infection. Such a vaccine might increase public confidence and result in better vaccine uptake. Meanwhile, the identification of more efficacious vaccination strategies with currently available vaccines reaching high vaccination coverage rates is required, including the vaccination of pregnant women (50).

## Author Contributions

SE proposed the project, coordinated the Study Group, and wrote the first draft of the study. PS, NF, and GF coordinated the sections of the project on Vaccines, Microbiology, and Immunology, respectively, giving a substantial scientific contribution. QH, PP, TT, MK, CR, CW, KF, IH, IL, KE, and MO gave a substantial scientific contribution. NP co-wrote the first draft of the manuscript and supervised the project. All authors approved the final submitted version of the manuscript.

### Conflict of Interest Statement

SE has received consultancy fees and independent research grants from GlaxoSmithKline group of companies, Sanofi-Pasteur, Merck, Vifor, and DMG. MK was member of advisory boards for GSK, Pfizer, Baxter, Novartis, Astra Zeneca, MedImmune, SPMSD, Sanofi, MSD, Jansen, and performed for these companies presentations during industry symposia. These activities were done as a service task for his employer. Personally, he did not receive any fees from companies. There was also no target agreement with his employer in this respect. MO has received an investigator initiative research grant from Merck to evaluate population perception on hexavalent vs. heptavalent vaccines in Chile. KF is on the vaccine advisory boards for Sanofi Pasteur and Seqiris and have received honoraria from AstraZeneca and Pfizer for giving talks. PP has received research funding from the National Institute for Health Research and from GlaxoSmithKline, and has received honorariums from Sanofi Pasteur and Pfizer. CR has received consultancy fees and independent research grants from GlaxoSmithKline group of companies, Sanofi-Pasteur MSD, Wyeth, Pfizer, Astra-Zeneca, and Astellas. The remaining authors declare that the research was conducted in the absence of any commercial or financial relationships that could be construed as a potential conflict of interest.
